# Improving hypertension management through pharmacist prescribing; the rural alberta clinical trial in optimizing hypertension (Rural RxACTION): trial design and methods

**DOI:** 10.1186/1748-5908-6-94

**Published:** 2011-08-11

**Authors:** Theresa L Charrois, Finlay A McAlister, Dale Cooney, Richard Lewanczuk, Michael R Kolber, Norman RC Campbell, Meagen Rosenthal, Sherilyn KD Houle, Ross T Tsuyuki

**Affiliations:** 1 School of Pharmacy, Curtin University, Perth, Western Australia, Australia; 2 Department of Medicine, University of Alberta, Edmonton, Alberta, Canada; 3 COMPRIS/EPICORE Centre, University of Alberta, Edmonton, Alberta, Canada; 4 Alberta College of Pharmacists, Edmonton, Alberta, Canada; 5 Faculty of Medicine, University of Calgary, Calgary, Alberta, Canada

## Abstract

**Background:**

Patients with hypertension continue to have less than optimal blood pressure control, with nearly one in five Canadian adults having hypertension. Pharmacist prescribing is gaining favor as a potential clinically efficacious and cost-effective means to improve both access and quality of care. With Alberta being the first province in Canada to have independent prescribing by pharmacists, it offers a unique opportunity to evaluate outcomes in patients who are prescribed antihypertensive therapy by pharmacists.

**Methods:**

The study is a randomized controlled trial of enhanced pharmacist care, with the unit of randomization being the patient. Participants will be randomized to enhanced pharmacist care (patient identification, assessment, education, close follow-up, and prescribing/titration of antihypertensive medications) or usual care. Participants are patients in rural Alberta with undiagnosed/uncontrolled blood pressure, as defined by the Canadian Hypertension Education Program. The primary outcome is the change in systolic blood pressure between baseline and 24 weeks in the enhanced-care versus usual-care arms. There are also three substudies running in conjunction with the project examining different remuneration models, investigating patient knowledge, and assessing health-resource utilization amongst patients in each group.

**Discussion:**

To date, one-third of the required sample size has been recruited. There are 15 communities and 17 pharmacists actively screening, recruiting, and following patients. This study will provide high-level evidence regarding pharmacist prescribing.

**Trial Registration:**

Clinicaltrials.gov NCT00878566.

## Background

### The problem

Patients with hypertension continue to have less than optimal blood pressure control. Nearly one in five Canadians adults--about 4.6 million people between the ages of 20 and 79--have high blood pressure [1]. Almost 50% of patients between the ages of 60 and 79 had a diagnosis of hypertension, with the highest rates of hypertension in elderly women [1,2]. In the United States and Canada, approximately 50% and 35%, respectively, of patients with hypertension are not controlled. These numbers increase to over 60% in patients with diabetes [3,4]. Although awareness and control of hypertension have improved significantly in the last 20 years, there still is a large gap in care.

### The problem in rural communities

Care of patients with chronic diseases living in rural communities is sometimes difficult given the shortage of rural physicians and the lack of access to specialty care and other healthcare professionals. There is also evidence to suggest that practice guidelines may be implemented to a lesser extent in rural communities [5,6]. This provides an excellent opportunity for a multidisciplinary shared-care approach to screening, diagnosis, management, and follow-up of patients with hypertension [7].

### Pharmacists helping to solve the problem

There have been a number of interventions by pharmacists to help close these gaps in hypertension management [8,9]. In a recently published study on physician and pharmacist collaboration in a community medical office, there was an adjusted mean change in systolic blood pressure (SBP) of -12.0 mm Hg at six months and an absolute improvement in blood pressure (BP) control rates of 34% (from 30% in usual care to 64%) [10]. A systematic review conducted by the same group [11] investigated the effects of team-based care for hypertension. Pharmacist recommendations to the physician decreased overall SBP by 9 mm Hg.

In a recently completed study by our group, Improving blood pressure management in patients with diabetes study (SCRIP*-HTN*), a community pharmacist and nurse intervention (in which the treatment team monitored patients' blood pressures and faxed guideline-concordant treatment recommendations to their primary care physicians if they were above target) produced a 5.6 mm Hg greater decrease in SBP over 24 weeks compared to usual care in patients with diabetes and poorly controlled blood pressure [12]. Despite the improvement in mean blood pressure, only half of the patients reached their blood pressure goal. One potential explanation for this is that traditional pharmacist care can only go so far with recommending appropriate therapy (*i.e*., there is a ceiling effect based on whether or not the physician prescribes the recommended therapy).

As healthcare costs worldwide increase, pharmacist prescribing is gaining favor as a potentially clinically efficacious and cost-effective means to improve both access and quality of care [13]. Pharmacist prescribing is defined differently depending on the region; it can range from prescribing by protocol to independent prescribing. The Canadian province of Alberta has been a pioneer in the area of independent pharmacist prescribing by being the first jurisdiction in Canada to enact legislation for such. Pharmacists in Alberta now have the ability to apply for additional prescribing authorization. This authorization allows them to prescribe for minor self-limited or self-diagnosed conditions, to modify a prescription written by another prescriber, or to undertake comprehensive drug therapy management, which can include independent initiation of drug therapies [14].

Pharmacist prescribing is a relatively recent innovation, and as such, there is little evidence about its clinical or economic impacts. For instance, work in the United Kingdom has focused generally on attitudes towards pharmacist prescribing and on the value of training programs for pharmacist prescribing authority [15-17].

With Alberta being the first province in Canada to allow independent prescribing by pharmacists, it offers a unique opportunity to evaluate outcomes in patients who are prescribed antihypertensive therapy by pharmacists.

## Methods

### Study overview

**Figure 1:**
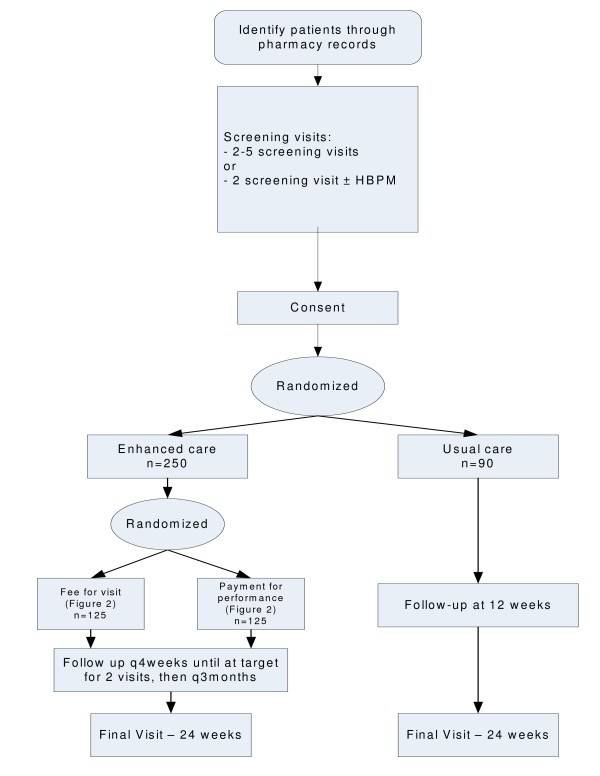
**Study Flow Diagram**. Flow diagram of randomization and follow-up of patients based on group assignment. Patients in enhanced care are followed up more closely until target blood pressure is reached. Patients randomized to usual care only have two follow-up visits. (HBPM = home blood pressure monitoring, q4 weeks = every 4 weeks, q3 months = every 3 months).

The study is a randomized controlled trial of enhanced pharmacist care, with the unit of randomization being the patient. Participants are randomized to either enhanced pharmacist care (patient identification, assessment, education, close follow-up, and prescribing/titration of antihypertensive medications) or usual care (Figure 1). Due to the nature of the intervention, blinding of patients and practitioners is not possible, although outcomes will be adjudicated in a blinded fashion.

The primary objective is to evaluate the effect of enhanced pharmacist care on SBP in patients with poorly controlled hypertension in the rural setting. Secondary objectives include the number of patients at their BP target at 24 weeks, the number of new antihypertensive medication starts, the number of antihypertensive dosage changes (increases and decreases in dose), the number of antihypertensive medication changes, and the number of new prescriptions for aspirin and cholesterol medications.

### Patients

Participants are patients in rural Alberta with undiagnosed or uncontrolled BP as defined by the Canadian Hypertension Education Program (CHEP) [18]. Pharmacists screen patients using the criteria in Table 1, as adapted from the CHEP guidelines for the management of hypertension by pharmacists [19].

**Table 1:** Screening parameters for pharmacists to identify potential participants

Screening Parameters	Details
Self-identified	

Screening days using validated devices	

Medication profiles	• Antidiabetes medications • Oral contraceptives • Antianginals • Antiplatelet agents • Cholesterol-lowering medications • Smoking cessation therapy • Treatments for erectile dysfunction

Patients with known cardio-, cerebro-, or peripheral vascular disease	

Patients with unfavorable cardiovascular risk factors	• Smokers • Overweight and obese • Increased age • Hyperlipidemia • Family history of cardiovascular disease

Referral from other healthcare professionals	

Patients are included if they meet one of the following criteria:

• Overall average (after two visits in undiagnosed patients without macrovascular target organ damage, diabetes [DM], or chronic kidney disease [CKD]) SBP ≥180 mm Hg or diastolic BP (DBP) ≥110 mm Hg

• Overall average (after two visits in patients with undiagnosed hypertension with macrovascular target organ damage) SBP ≥140 mm Hg or DBP ≥90 mm Hg

• An established diagnosis of hypertension above target BP, SBP ≥140 mm Hg (≥130 with DM or CKD), or DBP ≥90 mm Hg (≥80 with DM or CKD)

• Overall average (after five visits for those without macrovascular target organ damage, DM, or CKD and without an existing hypertension diagnosis) SBP ≥140 mm Hg or DBP ≥90 mm Hg

• Overall average (after seven days of twice-daily home BP monitoring for those without macrovascular target organ damage, DM, or CKD and without an existing hypertension diagnosis) SBP ≥135 mm Hg or DBP ≥85 mm Hg

These inclusion criteria are based on the CHEP criteria for the assessment of patients [18]. CKD is defined as patients with a glomerular filtration rate (as calculated by the Modification of Diet in Renal Disease equation) of < 60 mL/min for at least three months [20].

Patients are excluded if they are having a hypertensive urgency (defined as an SBP ≥200 mm Hg or DBP ≥130 mm Hg) or emergency, are unwilling to participate or sign the consent form, or if the patient is pregnant.

### Recruitment

Pharmacists actively screen for potential patients using the criteria in Table 1. Patients start by attending two screening visits, separated by two weeks. If their SBP is between 140-180 mm Hg and their DBP is between 90-110 mm Hg after the first two screening visits, they will be invited to either further evaluate their BP by home BP monitoring or have an additional three screening visits at the pharmacy. The home BP monitors will be lent to the patients by the pharmacists and are Canadian Hypertension Society approved devices.

### Randomization

**Figure 2:**
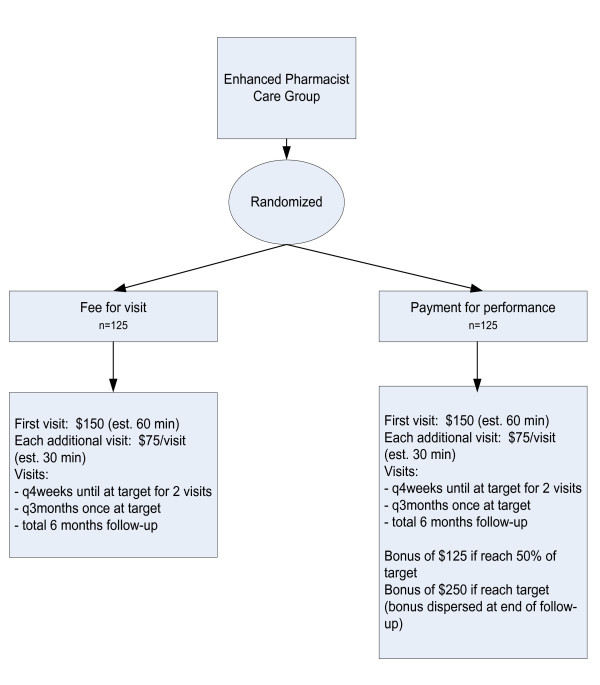
**Remuneration Flow Diagram**. Flow diagram of study design and group allocation for remuneration substudy, including payments schedule for pharmacists. (q4 weeks = every 4 weeks; q3 months = every 3 months).

After their screening is complete, patients will be invited to participate. Once the patient provides informed written consent, the participant is randomized (via a centralized secure website to ensure allocation concealment) in a 2:1 ratio to either enhanced pharmacist care or usual care (Figure 1) and enrolled by the pharmacist. The patients randomized to the enhanced pharmacist care group are further randomized to one of two payment structures for the pharmacist (fee for visit or payment for performance, Figure 2) in a 1:1 ratio. The randomization strategy employs variable blocked randomization stratified by study center.

### Intervention

The intervention (Table 2) is based upon the recommendations of CHEP [18,19]. Pharmacists assess patients with regards to BP control and cardiovascular risk reduction. The pharmacist also reviews the patient's current hypertensive therapy regimen and decides upon the options for improvement of BP control and implementation of these strategies. Pharmacists encourage adherence to antihypertensives. A fax is sent to the patient's primary care physician outlining the strategies discussed and implemented at the baseline visit. In some cases, depending on the proximity to the physicians, pharmacists may discuss the changes in person. Patients are followed at four-week intervals until achievement of target BP for two consecutive visits and, thereafter, at three-month intervals until study completion. To support pharmacists, there is "hotline" access to hypertension experts via email or telephone. Participating pharmacists submit questions that are either answered by a member of the study coordinating center or are forwarded to one of the hypertension experts on the steering committee.

**Table 2:** Components of enhanced pharmacist care

Intervention Component	Detailed Description
Blood pressure control	• Review current blood pressure control and treatment goals • Modify antihypertensive regimen as required (increase dose, additional antihypertensives, change administration schedule) • Order blood work as required with medication changes

Cardiovascular risk reduction	• Assessment of concomitant conditions and appropriate treatment (*e.g*., diabetes management, dyslipidemia, obesity)

Education	• Wallet card to record blood pressure readings, patient-related information from the Canadian Hypertension Education Program [17] • Lifestyle parameters for implementation (weight loss, salt intake, smoking cessation, alcohol intake, physical activity)

Collaboration with physicians	• Meeting with local physicians prior to study start to discuss collaboration and address any concerns, provide physicians with a communication package • Fax/letter regarding patient's enrollment in the study as well as notification regarding prescriptions

Adherence	• Simplified regimens, home blood pressure monitoring, and/or identification of adherence barriers

Close follow-up	• Every four weeks until patient reaches target for two consecutive visits, then every three months

### Control group

Patients randomized to the control group receive usual care (which is actually more than typical "usual care" in these settings): a wallet card for BP readings and a pamphlet on BP. Patients are seen at 12 weeks for interim BP measurement only. Both groups of patients are seen at 24 weeks for final BP measurements. If the pharmacist has a concern regarding control patients' BP during the study, they are to make recommendations to the physician and refer patient to physician, as appropriate. Usual-care patients will be offered intervention care at the end of the follow-up; as such, prescribing by the pharmacist for control patients can occur after 24 weeks.

### Outcomes

The primary outcome is the change in SBP between baseline and 24 weeks in the enhanced-care versus usual-care arms. All BP measurements are made using BpTRU^® ^(BpTRU Medical Devices, Coquitlam, British Colombia, Canada), an approved and validated measuring instrument, as per CHEP [18]. Secondary outcomes include the number of patients at their BP target at 24 weeks, the number of new antihypertensive medication starts, the number of antihypertensive dosage changes (dose increases or decreases), the number of antihypertensive medication changes, and the number of new prescriptions for aspirin and cholesterol medications. Pharmacists will be asked to participate in end-of-study focus groups to determine their opinions on the different remuneration models.

### Setting

Pharmacists and pharmacies are selected based on their current practice model, size, and location of the community (population of < 20,000 and located more than one-hour driving distance from Edmonton and Calgary) and willingness to apply for additional prescribing authorization.

Currently we have 15 sites involved in the study, with two sites that have multiple pharmacists participating. The majority of sites are community pharmacies, but sites also include pharmacists in primary care networks and hospital-based pharmacists who provide some outpatient services. All study data are collected in the pharmacy and faxed to the study office using unique patient identifiers to conceal the identity of the patients.

### Prescribing authorization

Pharmacists participating in the study must be authorized to prescribe as defined by the Health Professions Act in Alberta [14]. In order to do this, pharmacists must have at least two years of patient-care experience and have successfully completed an application process. The study team assists pharmacists in implementing documentation systems and accruing the information required to complete their application. To that end, pharmacists attend educational seminars with local hypertension experts that help fulfill the requirement for demonstrating continued education in the area of interest. In addition, pharmacists recruit a run-in of at least three patients and conduct all elements of the intervention except the prescribing portion (patient identification, assessment, education, recommendations to physician). This run-in can serve as the basis for pharmacists to ensure that they routinely perform all the key activities that are required for additional prescribing authorization

### Sample size and statistical analysis

Based on a two-sample, two-sided *t*-test, a sample size of 162 will provide 80% power to detect a minimally important clinical difference (MCID) of 8 mm Hg between intervention and usual-care groups, with a projected standard deviation of 18 in each group [12]. In the comparison of different payment models within the intervention arm, an alpha of 0.10 was used for a total of 224 patients to detect a 6 mm Hg MCID between the fee-for-visit group and the payment-for-performance group.

Assuming a drop-out or loss to follow-up rate of 10%, the sample size has been increased to 250 in the intervention group (125 in fee for service and 125 in payment for performance) and 90 in the usual-care group, for a total sample size of 340.

All analyses will be intention to treat. A comparison of baseline characteristics will be performed using *t*-tests or nonparametric Wilcoxon for continuous variables and chi-squared test for categorical variables. In order to account for any imbalance in baseline characteristics, the test for differences in change in SBP between intervention and usual-care groups will be adjusted for using multiple regression models (or analysis of covariance models). The problem of missing values will be checked for nonrandomness, and appropriate imputation methods will be applied when needed. All analyses will be carried out using SAS 9.1.3 (SAS Institute, Cary, NC, USA).

### Research Ethics

Ethics approval for the study has been obtained from the University of Alberta Health Research Ethics Board.

### Substudies

#### Examining the effect of different remuneration models for pharmacists

Current remuneration for community pharmacists in Alberta is based on a per-prescription fee. However, with the advancement of pharmacy services to a patient-centered care model, research into other payment models is a priority. A recent review of remuneration systems for pharmacy services demonstrated a wide variety of models in place, with little analysis of outcomes and sustainability [21].

The objective of this substudy is to evaluate the clinical effect of two pharmacist remuneration models: a payment-for-performance model versus a fee-for-visit model.

Patients randomized into the intervention arm of the study are further randomized in a 1:1 ratio into either payment-for-performance or fee-for-visit remuneration models for the pharmacist. For those patients randomized into the fee-for-visit remuneration model, the pharmacist providing care receives a payment of $150 CDN for the first visit (expected to last approximately 60 minutes) and $75 per visit thereafter (expected to last approximately 20 minutes, plus 10 minutes for documentation), up to a maximum of six visits (Figure 2). For patients randomized into the payment-for-performance remuneration model, the pharmacist providing care receives $150 for the initial visit and $75 for each additional visit as outlined above, and also receives a bonus of $125 if the patient reaches 50% of their target (*i.e*., 50% of the change from baseline needed to reach target) and/or $250 if the patient reaches their target BP for two consecutive visits.

The primary outcome for this substudy is a comparison of SBP reduction achieved between patients in the payment-for-performance remuneration group versus the fee-for-visit remuneration group at six-months follow-up.

#### Understanding effects of enhanced pharmacist care on patient's hypertension knowledge

There is a potential lack of knowledge translation from clinicians to patients. Recently, a number of studies have been conducted assessing the knowledge of hypertensive patients regarding their condition. These studies have shown that most patients have little if any knowledge regarding their hypertension or the potential long-term sequelae of hypertension [22-26]. One study that specifically focused on patient attitudes towards hypertension found that only 35% of hypertensive patients felt that high BP was a serious health concern, and over 35% also felt that high BP was unavoidable [25]. Patient knowledge of hypertension, its causes and symptoms, and the seriousness of having uncontrolled BP must be improved upon.

The objective of this substudy is to evaluate the effect of enhanced pharmacist care on patient knowledge. Two surveys are administered to all patients enrolled in the study. One survey is to be completed at the time of enrollment, while the other is an exit survey. Survey questions are designed to determine patients' level of knowledge regarding hypertension and blood pressure [25], as well as patients' level of understanding and experience with pharmacy interventions [27,28].

We plan to compare hypertension knowledge at baseline and at follow-up between the control and intervention patients. Other outcomes include a comparison of the knowledge levels of patients in the control and intervention groups regarding pharmacists' contribution to patient care and any differences in knowledge between those patients randomized to the payment-for- performance versus fee-for-visit intervention models. These analyses will only be completed for those patients who complete the intervention.

#### Resource utilization

There is little evidence about the economic impacts of pharmacist prescribing. Yet, if BP is reduced in a sustained fashion, this will reduce strokes and other cardiovascular events, which would likely have a significant economic impact. In a systematic review of pharmacist prescribing in the United Kingdom, none of the included studies had an economic component [29]. A retrospective review of supplementary pharmacist prescribing in the United Kingdom showed that between 2004 and 2006, pharmacist prescribing accounted for 0.004% of all prescriptions and 0.003% of prescribing costs [30]. This analysis was limited to prescription costs only; there was no consideration of costs related to pharmacist time, healthcare utilization, or other patient-related costs (*e.g*., travel, home BP monitoring).

The primary objective of this substudy is to evaluate the effect of enhanced pharmacist care on healthcare utilization (hospitalization, emergency room visits, physician visits, use of laboratory testing, use of antihypertensive and other vascular risk-reduction medications). Data will be obtained through Alberta Health Services and Alberta Health and Wellness regarding physician visits and hospitalizations.

### Study organization and management

The study steering committee consists of pharmacists, researchers, a family physician in rural practice, and physicians with expertise in hypertension. The steering committee is involved in study design, education of pharmacists, and liaising with local physician groups and is available for consultation for difficult cases. The study coordination, including randomization, database design, data entry, and project management, is done centrally through COMPRIS/EPICORE Centre, a nonprofit academic research organization http://www.epicore.ualberta.ca.

### Investigator meetings

In April 2009, the first investigator meeting and training session was held. It consisted of a didactic component on the current guidelines for the treatment of hypertension and was followed by small group discussions on patient cases, study procedures, patient assessment, and applying for additional prescribing authorization.

Additional pharmacists were invited to participate in the study, and another investigator meeting was held in September 2010 as an update to pharmacists who were already enrolled and a welcome to the new pharmacists.

An interim meeting will also be held once half the required patients are accrued.

### Site visits

The sites are spread across the province, with the two farthest points being over 1,000 kilometers apart. In order to offer continued and ongoing support to the pharmacists, site visits are scheduled during recruitment. The first wave of site visits was conducted during May 2010, with each site being visited by one or two study team members. Information regarding community-specific recruitment strategies was provided to each pharmacist.

### Ongoing support

In order to maintain pharmacist engagement in the study, the study team offers various methods of support. Firstly, monthly newsletters are sent to all sites as well as study sponsors. The newsletters contain study updates, specifically, enrollment to date; clinical pearls; study form reminders; cases; and summaries of recent research. The newsletter format is short (usually three to four pages), and therefore, additional information is often posted on the study website for pharmacists who want further information. Secondly, the study website includes posted information, notices, and discussion boards where pharmacists can post questions and have their colleagues respond. Finally, teleconferences are scheduled monthly, and all pharmacists are invited to participate. The teleconferences are held on rotating days and in the evening, to help accommodate any other commitments the pharmacists may have. An agenda is circulated prior to the teleconference, along with any additional information that may be discussed (a recent study, patient cases, etc.). The study pharmacists can also submit questions or cases that they would like help with. They present the information to the other pharmacists on the call and discussion ensues. Of all the ongoing support mechanisms in place, the pharmacists use the teleconferences most frequently.

### Study status

To date, one-third of the required sample size has been recruited. There are 15 sites and 17 pharmacists actively screening, recruiting, and following patients.

## Discussion

The study design randomizes patients to enhanced pharmacist care or usual care at the patient level rather than at the pharmacist level. Because of the additional workload inherent in enhanced pharmacist care (including provision of lifestyle counselling, prescribing/titration of antihypertensive medications, and more frequent follow-up) a cluster approach was not seen to be feasible for pharmacists who would have been randomized to enhanced care by this approach. In addition, since all pharmacists involved in the study have additional prescribing authorization, it would not be acceptable for them to be randomized to usual care only. However, the possibility for contamination of the sample is acknowledged. Such contamination may affect the quality of care provided to usual-care patients by each pharmacist and may also affect the pharmacists' responses to the two remuneration schemes since pharmacists will potentially be subject to receiving different payments among their enhanced-care cohort of patients. While contamination may occur, it will bias results towards the null hypothesis; therefore, if a significant difference is found, the result will be more robust.

## Competing interests

TLC, MR, RL, MRK, DC, SKDH, and FAM declare that they have no competing interests.

NRCC does not have any financial interests that relate to pharmacists caring for people with hypertension. He has talked for Sanofi-aventis, Bayer, Biovail, Bristol-Myers Squibb, and Boehringer Ingelhiem; received travel support from Bayer, Schering-Plough, and Boehringer Ingelhiem; and is on advisory boards for Novartis, Schering-Plough, Bristol-Myers Squibb, Sanofi-aventis, and Pfizer. Nonfinancial interests include being the Canadian Institutes of Health Research (CIHR) Canada Chair in Hypertension Prevention and Control.

RTT has received unrestricted research funding from Merck, AstraZeneca, Sanofi-aventis, ManthaMed, Bristol-Myers Squibb, Apotex, Bayer, and Medtronic. He has received speaking honoraria from Merck, Novartis, Bayer, Sanofi-aventis, and Bristol-Myers Squibb.

## Authors' contributions

All authors have made substantial contributions to the study conception and design, acquisition of data, and development/editing of the manuscript and have given final approval of the version to be published.
